# A Scientific Assessment of Sociodemographic Factors, Physical Activity Level, and Nutritional Knowledge as Determinants of Dietary Quality among Indo-Mauritian Women

**DOI:** 10.1155/2013/572132

**Published:** 2013-05-25

**Authors:** Yashvee Dunneram, Rajesh Jeewon

**Affiliations:** Department of Health Sciences, Faculty of Science, University of Mauritius, Reduit, Mauritius

## Abstract

A healthy diet is of particular concern throughout the life of women to avoid many chronic illnesses especially during their 30s to 50s. There are published data on dietary quality and its determinants among women, but there is a lack of similar data regarding women in Mauritius. This study aimed to investigate the association between age and dietary quality in relation to sociodemographic factors, physical activity level (PAL) and nutritional knowledge (NK). A survey-based study was conducted in 2012 among Indo-Mauritian women including 117 young (21.35 ± 1.98), 160 reaching middle age (34.02 ± 5.09) and 50 middle-aged (37.85 ± 8.32). Validated questionnaires were used to elicit information on the determinants. A food frequency table consisting of 18 food items was used to assess dietary quality. Univariate and multivariate analyses were used to determine the association between various factors and dietary quality. The mean dietary score of middle-aged women (18.70 ± 2.67) was closer to recommended dietary guidelines compared to young women (17.22 ± 3.40), and women reaching middle age (17.55 ± 3.29). Educational level, PAL, NK, and age were main determinants of dietary quality among Indo-Mauritian women (*P* < 0.05). Younger women with low educational level, PAL, and NK are at risk of poor dietary quality.

## 1. Introduction

Mauritius is a middle-income country with a per capita Gross Domestic Product of US$15,600 PPP in 2012 [1]. It is a multiethnic nation [2] which has experienced rapid economic growth since the 1980s, with amplification in disposable income followed by changes in food consumption and lifestyle patterns similar to the globally observed trend [3, 4]. This shift in nutrition habits has been illustrated by an increase in the consumption of meat, eggs, dairy products, oils, and fats which has led to the rapid increase in the occurrence of overweight, obesity, and associated noncommunicable diseases such as diabetes and cardiovascular problems. Few studies have documented the Mauritian dietary quality [4], but there is a lack of published data on the dietary of women in relation to various determinants.

Consuming a healthy diet and having access to a nutritious supply of food are important to good health, as good nutrition is a key factor in the overall health and wellbeing of women [5]. Ensuring proper nutrition among women has several optimistic outcomes because healthy women can fulfil their multiple roles, generating income, ensuring their families' nutrition, and having healthier children, and thus help progress countries' socioeconomic development [6]. A more comprehensive study of women's dietary patterns is warranted for a number of reasons. Dietary habits have a key role in weight management and successful weight loss, but regulating and sustaining it is quite complex, especially over a long term [7]. A recent longitudinal study over a period of 16 years found that over half of the women in USA gained weight [8]. Additionally, dietary factors especially during early life may lead to many of the chronic health conditions experience, by women in their middle age and later years such as cardiovascular disease, type II diabetes, osteoporosis, and certain cancers [9]. This can be supported with data from recent studies which reported a higher prevalence of chronic health conditions (joint problems, stroke, heart disease, diabetes, pulmonary disease, hypertension, or cancer) among women as compared to men [10–12]. McCullough et al. [13] demonstrated that higher dietary scores in terms of meeting dietary guidelines were associated with significant reductions in risk of major chronic disease in women. Therefore, behaviour to develop healthful dietary habits should start early and continue through life [14].

It has been well documented that dietary pattern is usually complex and determined by several interrelated factors, including age, sex [15], sociodemographic characteristics—education, occupation, income, ethnicity, and knowledge and attitude related to diet and health [16]. Research across several countries has shown that healthier diets composed of lean meats and fresh fruits and vegetables tend to cost more as compared to unhealthy diets high in refined grains, added sugars, and added fats [17], and thus people from the higher socioeconomic segment are more consistent with recommended dietary guidelines [18]. Apart from income which determines types of food consumed, educational level and occupation also influence dietary quality. This can be explained by the fact that education may influence diet through the comprehension of nutritional information and food labeling, while income decides affordability of preferred food, and occupation may impinge on dietary quality during work time, social environment, and to the lifestyle of colleagues [19].

Unhealthy behaviours such as smoking, alcohol consumption, and low physical activity level have also been shown to be factors influencing dietary quality [20, 21]. Data on the association between dietary pattern and physical activity as well as smoking indicate that nonsmokers [22] and women with higher physical activity level [23] had a food pattern more in line with dietary allowances in terms of decreased consumption of total calories, total fat, saturated fat, protein, and cholesterol [24, 25]. Nutritional knowledge is another major determinant of dietary quality among adults [14, 26]. Yet, many studies have been unable to demonstrate a clear link between nutritional knowledge and dietary behaviour [26–28].

Hence, factors influencing dietary behaviours need to be better understood to develop effective nutrition interventions adapted to individuals to improve their dietary quality [29] and to support nutrition programs or policies [9]. In this study, we determined the association between age and dietary quality in relation to sociodemographic factors, physical activity level (PAL), and nutritional knowledge (NK) among women.

## 2. Materials and Methods

The sample size was calculated using the “Small-Sample Techniques” from the research division of the National Education Association [30]. Using a stratified random sampling method, 384 participants were approached but due to refusal to participation, pregnancy, and some women being on medication or special diet, 57 of them were excluded. Out of the total 327 women, 117 women were classified as young (18–25 years old), 160 as reaching middle age (26–44 years old), and the remaining 50 as middle-aged women (46–55 years old). Participants were recruited from several community and social centres across Mauritius. After obtaining informed consent, survey was carried out in 2012. Research has been granted approval by the University Research Ethics Committee.

### 2.1. Data Collection

A face to face interview was conducted to investigate the dietary habits, physical activity level and nutritional knowledge of the participants as well as to determine their ethnic group, sociodemographic factors—income level, education level, employment status, marital status, and family structure (nuclear and extended family) [31], and BMI.

A food frequency table (FFT), consisting of 18 food items and beverages, was adapted from that of Fokeena and Jeewon [32] to assess participants' dietary quality. A score was generated for the consumption frequencies of each food items (more than once daily = 4; once daily = 3; once or more per week = 2; once or more per month = 1; rarely/never = 0) to compare consumption pattern of the 18 food items between young and middle-aged women. Moreover, to evaluate whether the participants meet the recommended dietary guidelines, dietary guidelines for the prevention of noncommunicable diseases for Mauritian adults [33] and MyPlate for moms developed by the California Department of Public Health [34], depending on participants' consumption frequency of 18 food items as per the FFT, and other meal patterns such as type of milk used (full cream milk = 0; semiskimmed/skimmed milk = 1), number of meals per week (having breakfast/lunch/dinner everyday = 1; skipping breakfast/lunch/dinner = 0), total number of meals during weekends (3 meals = 1; 2 or 1 meal = 0), and having balanced meals, it was determined whether dietary guidelines were being followed (1 = meet dietary guideline; 0 = do not meet dietary guideline), which accounted to a total score of 26. Participants' diet was assessed as being balanced or not, using the MyPlate for moms [34].

For anthropometric measurements, height was recorded using a measuring tape, with the individual standing straight against a wall, with the heels, buttocks, and shoulders touching the wall. Weight was measured using normal weighing scales with the individual wearing light clothes and no shoes [35]. BMI was then calculated as weight in kilograms divided by height squared in meters (kg/m^2^) and categorized as underweight (BMI < 18.5 kg/m^2^), normal (BMI 18.5–24.9 kg/m^2^), overweight (BMI 25.0–29.9 kg/m^2^), and obese (BMI ≥ 30.0 kg/m^2^) [36, 37]. 

Physical activity level (PAL) was assessed using the shortened version of the self-reported International Physical Activity Questionnaire (IPAQ) [38] which has been previously tested for its validity and reliability in 12 countries [39]. PAL was calculated in metabolic equivalents (MET)—minute per week—and categorized into low (<500 MET), moderate (500–2500 MET), and high (>2500) PAL [38]. 

Questions on nutritional knowledge were adapted from the General Nutrition Knowledge Questionnaire for adults developed by Parmenter and Wardle [27]. A score of 1 was assigned to a good answer and 0 otherwise which was assessed on a total score of 25, whereby higher values on the score indicate higher nutritional knowledge. The total score was divided into tertiles, where the lowest one referred to “insufficient nutritional knowledge,” the medium one referred to “quite good nutritional knowledges” and the highest one referred to “good nutritional knowledge” [29].

### 2.2. Statistical Analysis

Data were analysed using the Statistical Package for the Social Sciences (version 17.0). Chi square test was conducted to determine participants' sociodemographic characteristics, BMI, PAL, and NK in relation to age group. Pearson product-moment correlation was used to assess the relationship between age and BMI. One-way analysis of variance was used to compare the consumption frequency of the 18 food items and dietary quality of young and middle-aged women. Two-way analysis of variance was carried out to associate different sociodemographic characteristics (income level, education level, employment status, marital status, and family structure), physical activity level, and nutritional knowledge with diet quality in relation to age. A multiple regression model was used to investigate the combined effects of age, income level, educational level, employment status, marital status, family structure, PAL, and nutritional knowledge on diet quality. Statistical significance was achieved when *P* < 0.05.

## 3. Results

### 3.1. Sample

The sociodemographic characteristics of the sample are shown in Table 2. The mean age and BMI mean value of the sample of young women (18–25 years) were 21.35 ± 1.98 and 21.35 ± 3.74, respectively. For those reaching middle age (26–44 years), the mean age was 34.02 ± 5.09 and BMI mean value was 23.95 ± 3.66. On the other hand, the mean age and BMI mean value of the sample of middle-aged women (45–55 years) were 50.12 ± 3.07 and 24.73 ± 3.42, respectively. A medium, positive correlation was found between age and BMI (*r* = 0.37, *n* = 327, *P* < 0.001).

### 3.2. Age and Dietary Habits

#### 3.2.1. Dietary Guidelines Met by Each Age Group

The mean dietary score was higher for middle-aged women (18.70 ± 2.67) as compared to those reaching middle age (17.55 ± 3.29) and the young women (17.22 ± 3.40) (*P* < 0.05). 

#### 3.2.2. Consumption of Food Items and Dietary Pattern for Each Age Group

Consumption of breakfast cereals (*P* = 0.332), whole-grain cereals (*P* < 0.05), beans (*P* < 0.05), dairy products (*P* = 0.309), red meat (*P* = 0.475), lean meat (*P* = 0.314), eggs (*P* = 0.651), processed meat (*P* < 0.01), fish (*P* = 0.156), sweetened foods (*P* < 0.01), nonalcoholic fizzy drinks (*P* < 0.01), and alcoholic drinks (*P* = 0.820) was higher among young women and those reaching middle age, while refined cereals (*P* = 0.751), fruits (*P* = 0.299), cooked vegetables (*P* = 0.317), raw salad (*P* = 0.734), fried (*P* < 0.05), and fast foods (*P* < 0.01) were mostly consumed by the middle-aged women. The majority of young women (51.3%), those reaching middle age (50.0%), and middle-aged (56.0%) women consumed mostly full cream milk (*P* = 0.364). Meal skipping was rather infrequent among the participants both during weekdays and weekends (*P* > 0.05). Moreover, the majority of participants from both age groups had balanced meals (see Table 1). 

### 3.3. Sociodemographic Differences in Dietary Quality

The mean dietary score was higher among middle-aged women with the higher education level (*P* < 0.01), higher income level (*P* > 0.05), and middle-aged women who were part-time or full-time employed (*P* > 0.05) as compared to younger women with similar education level, income level and employment status. Marital status and family structure did not have a statistically significant influence on mean dietary score (*P* > 0.05).

### 3.4. Physical Activity Level and Dietary Quality

The majority of young women (67.5%, MET = 1372.46 ± 1268.58), women reaching middle age (56.9%, MET = 1775.62 ± 1521.86) and middle-aged women (50.0%, MET = 2043.78 ± 1792.66) had moderate physical activity level (PAL) (*P* < 0.05). Only 11.1% of young women, 194%, women reaching middle age and 30.0% of middle-aged women had a very active lifestyle while around one-third (21.4%; 23.8%; 20.0%) of them, respectively, had a low physical activity level, not consistent with a healthy lifestyle. In addition, our results (Table 3) showed that the mean dietary score was higher among middle-aged women with higher PAL as compared to young women and women reaching middle age with high PAL (*P* < 0.01).

### 3.5. Nutritional Knowledge and Dietary Quality

Most of the participants of all three age groups had good nutritional knowledge (NK) (65.8% young women; 63.8% women reaching middle age; 62.0% middle-aged women; *P* > 0.05). Younger women (17.74 ± 3.73) and women reaching middle age (17.52 ± 4.22) had higher mean nutritional knowledge score than middle-aged women (16.56 ± 4.18). However, two-way between-groups analysis of variance (Table 3) showed that middle-aged women with good NK (19.19 ± 2.57) had higher mean dietary score as compared to the two other age groups (*P* < 0.01).

### 3.6. Age, Sociodemographic Factors (Income Level, Education Level, Employment Status, Marital Status, and Family Structure), PAL, Nutritional Knowledge, and Diet Quality

In order to ascertain whether age group, income level, education level, employment status, marital status, family structure, PAL, and nutritional knowledge were all having separate effects on diet quality, these variables were subjected to a standard multiple regression model. Results showed that nutritional knowledge followed by age, PAL, education level, family structure, employment status, marital status, and income level had the highest influence on diet quality. But only age, PAL, and nutritional knowledge had significant independent effects at the 0.01 level, and the effect of education level was significant at the 0.05 level, while the other variables did not have significant impact (Table 4). Together these four variables accounted for 7.8% of the variance in diet quality (*P* < 0.01).

## 4. Discussion

Based on population statistics in Mauritius [40], the number of working and educated females has been increasing over the past years, which had led to higher income level and thus, significant change in dietary quality has been observed. This study also demonstrates that middle-aged women and women with higher PAL as well as good NK had better dietary quality. The results reported herein provide substantial data on various factors that can influence dietary quality of women which can be useful when dealing with health-related issues especially chronic diseases among younger women and women with low income level, educational level, PAL, and NK.

### 4.1. Dietary Quality and BMI of Participants

There is a statistically significant increase in BMI with increasing age. Most women of the age groups (58.1% young women, 55.6% reaching middle age, and 52.0% middle-aged women) are in the normal range of values (18.5–24.9 kg/m^2^), while the incidence of obesity among women was low (1.7% young women, 5.0% women reaching middle age, and 8.0% middle-aged women). Yet, there was a significant portion of overweight, subjects among the groups with 17.1% of overweight young women, 34.4% women reaching middle age and 36.0% of middle-aged women. On the other hand, only a few middle-aged women (4.0%) and those reaching middle age (5.0%) were found to be underweight while a considerable 23.1% of women aged between 18–25 years were underweight. This data is in agreement with the National Non-Communicable Diseases Survey 2009 [41] conducted in Mauritius, where 35.1% of women were reported to be overweight. The higher incidence of overweight among middle-aged women reported herein can be explained by the fact that most of them had moderate PAL, in addition to quite frequent consumption of foods such as refined cereals, fried and fast foods, and consumption of full cream milk as compared to younger women.

Based on the dietary scores and FFT, middle-aged women had healthy diet compared to young women. This is supported by the higher consumption of fruits, cooked vegetables, raw salad and low consumption of red meat, processed meat, sweetened foods, nonalcoholic fizzy drinks and alcoholic drinks. Yet, consumption of fried foods and fast foods was rather high, while consumption of breakfast cereals, whole-grain cereals, and fish was low. The results are consistent with a study carried out by Dobson et al. [20] who also found more frequent consumption of fruits and vegetables among middle-aged women. However, they also reported lower consumption fried and fast foods among middle-aged women. 

Middle-aged women were also closer to recommended dietary scores. Koch and Pokorn [42] also reported better nutritional habits among middle-aged adults above 45 years in Slovenia. Healthier dietary quality among middle-aged women can be explained by the fact that since many women in their middle age may experience chronic health conditions such as cardiovascular disease, type II diabetes, osteoporosis and certain cancers [9] or in order to prevent these conditions, these women tend to adopt healthier eating patterns.

### 4.2. Correlation between Sociodemographic Factors and Dietary Quality

The commonly used indicators of social class are education, income, and occupation. A large number of epidemiological studies have associated diet quality with socioeconomic status [15, 19]. Our result showed that only one of the socioeconomic indicators (education level) had statistically significant association with diet quality in relation to age. However, a recent study reported a positive influence of all three indicators on dietary quality [19]. In addition, Lallukka et al. [43] also indicated the contribution of higher occupational class to healthy food habits among women. Evidence from several studies across Europe, USA and Australia which has consistently shown that those of higher social class (generally defined using education as an indicator, rather than income or occupation) have healthier diets (eating more fruit, vegetables, and low-fat or skimmed milk, as well as fewer fats and oils, and less meat) and consuming diets that are closer to the dietary recommendations [18]. Nutritional research has advocated that since diets high in refined grains, added sugars, and added fats usually cost less than healthier diets composed of lean meats and fresh fruits and vegetables, the poorer segment of the population is more exposed to unhealthy dietary choices [17]. 

In this study, no significant relationships were found between dietary quality and marital status as well as family structure. Conversely, few studies have shown a significant influence of these two factors on dietary quality. These studies have reported healthier dietary habits among married people [44] and those living in extended families as result of a supportive environment [31]. These disparities can be explained by the fact that participants in our study were mostly single or married and lived in nuclear families.

### 4.3. Association of Physical Activity Level (PAL) and Nutritional Knowledge (NK) with Dietary Quality

In the three age categories, the respondents mostly had moderate physical activity level. Yet, a larger proportion of middle-aged women were found to engage in vigorous PAL compared to their younger counterparts. Coleman et al. [45] found that young women with lower PAL reported less positive images of “sport,” negative perceptions of their own ability, frail motivations and personal choice to engage in activities, and no supporting influence of their friends and family as well as the detrimental impact of life transitions such as moving from college to full-time employment decreasing participation rate in physical activities. As mentioned earlier, risks of chronic conditions increase with increasing age [9], and middle-aged women are very concerned about their body image which accounts for higher PAL among them [46, 47] as compared to younger women. Data from another study has shown that high PAL among middle-aged women mitigates the effects of aging [48]. Results reported herein also showed that diet quality was significantly influenced by PAL. Our result can be substantiated with those from Midhet et al. [49] who also reported similar finding.

As far as NK is concerned, young women (17.74 ± 3.73) had higher mean NK score than middle-aged (17.29 ± 4.22) women. However, middle-aged women with good NK (18.40 ± 3.06) had higher mean dietary score as compared to younger women with good NK (17.53 ± 3.41) (*P* < 0.01). A plausible explanation is possibly a lack of interest in health care issues among younger age groups, while nutrition-related matters become more salient for middle-aged people as they become increasingly aware of diseases related to diet, and they are also more likely to seek for dietary information to ensure that their children are eating healthily [50]. A study among young and middle-aged Belgian women also found that women who had better nutritional knowledge demonstrated better dietary behaviour [51]. However, data reported on the association between NK and diet quality are not always consistent. For instance, Sharma et al. [14] found that the intake of several food groups increased with increased NK except for fruits and vegetables, while another study reported that an increase in NK was associated with a significant rise in consumption of vegetables and fruit, but no differences were seen for other dietary indicators [51].

There are several limitations in this study. Most of the data source was self-reported and thus more prone to bias. Food frequency questionnaires are good measures of long term diet; however, they have several drawbacks such as they contain a substantial amount of measurement error and there may be possible overreporting of “healthy” foods (e.g., fruit and vegetables) [52–54]. The sample also consisted of fewer middle-aged women as compared to younger women which were attributed to their unwillingness to participate in the study.

## 5. Conclusion

Our study demonstrates important determinants of dietary quality of Indo-Mauritian women, which include age, education level, PAL, and NK. Middle-aged Indo-Mauritian women with higher educational level, income level, employment status, PAL and NK were closer to recommended dietary guidelines compared to younger women. One advantage of this study is that it provides information on the determinants of women's dietary quality which can be used to be considered in tailoring nutrition educational programs and intervention programs. Implications of this study may contribute to the mounting evidence base required to establish or reinforce nutrition policies developed mainly by the Ministry of Health and Quality of Life.

## Figures and Tables

**Table 1:** Consumption frequency scores and dietary guideline scores for each food group.

Food groups	Consumption frequencies	Frequency Scores	Recommended dietary guidelines	Dietary guideline scores
(1) Whole-grain cereals (2) Fibre-rich breakfast cereals (3) Refined cereals	More than once daily	4	*Eat mostly whole grains	
	Once daily	3		1
	Once or more per week	2		
	Once or more per month	1		0
	Rarely/never	0		

(4) Fruits (5) Raw salad (6) Cooked vegetables	More than once daily	4	*Add colour with fruits **Eat more fruits and vegetables	
	Once daily	3		1
	Once or more per week	2		
	Once or more per month	1		0
	Rarely/never	0		

(7) Beans or pulses	More than once daily	4	*Eat vegetable protein daily **Eat more pulses and legumes	
	Once daily	3		1
	Once or more per week	2		
	Once or more per month	1		0
	Rarely/never	0		

(8) Dairy products	More than once daily	4	*Enjoy calcium-rich foods	
	Once daily	3		1
	Once or more per week	2		
	Once or more per month	1		0
	Rarely/never	0		

(9) Red meat (10) Lean meat (11) Eggs (12) Processed meat	More than once daily	4	**Eat foods that are low in fat, especially saturated fat	
	Once daily	3		0
	Once or more per week	2		
	Once or more per month	1		1
	Rarely/never	0		

(13) Fish	More than once daily	4	*Choose healthy protein **Eat foods that are low in fat, especially saturated fat	
	Once daily	3		1
	Once or more per week	2		
	Once or more per month	1		0
	Rarely/never	0		

(14) Sweetened foods (15) Nonalcoholic fizzy drinks	More than once daily	4	**Limit your intake of added sugars from beverages and foods	
	Once daily	3		0
	Once or more per week	2		
	Once or more per month	1		1
	Rarely/never	0		

(16) Fast foods (17) Fried foods	More than once daily	4	**Choose and prepare foods with less salt **Eat foods that are low in fat, especially saturated fat	
	Once daily	3		0
	Once or more per week	2		
	Once or more per month	1		1
	Rarely/never	0		

(18) Alcoholic drinks	More than once daily	4	*Do not drink alcohol when you may become pregnant **If you drink alcohol, do so in moderation	
	Once daily	3		0
	Once or more per week	2		
	Once or more per month	1		1
	Rarely/never	0		

*CDPH: California Department of Public Health; **MOHQL: Ministry of Health and Quality of Life (Mauritius).

**Table 2:** Percentage distribution of sample sociodemographic characteristics.

Demographic characteristics	18–25 years (*n* = 117)	26–44 (*n* = 160)	45–55 years (*n* = 50)	*P* value
Area of residence				
Urban	**52.1**	40.0	34.0	0.199
Rural	47.9	**60.0**	**66.0**	
Ethnic group				
Indo-Mauritian	**94.0**	**80.6**	**90.0**	0.050*
Afro-Mauritian	6.0	15.6	8.0	
Sino-Mauritian	0.0	2.5	2.0	
Europids	0.0	1.3	0.0	
Marital status				
Single	**85.5**	11.9	6.0	0.000**
Married	14.5	**86.3**	**88.0**	
Divorced/separated	0.0	1.3	2.0	
Widowed	0.0	0.6	4.0	
Cohabitation	0.0	0.0	0.0	
Family structure				
Nuclear	**81.2**	**79.4**	**72.0**	0.402
Extended	18.8	20.6	28.0	
Employment status				
Full time	29.1	0.0	34.0	0.000**
Part time	4.3	16.3	2.0	
Self-employed	3.4	11.9	14.0	
Housewife	3.4	4.4	**50.0**	
Student	**59.8**	**67.5**	0.0	
Income level				
Low	20.5	16.9	22.0	0.790
Moderate	**44.4**	**49.4**	**50.0**	
High	35.0	33.8	28.0	
Education level				
Low	0.9	6.9	26.0	0.000**
Medium	5.1	13.8	18.0	
High	**94.0**	**79.4**	**56.0**	

*P < 0.05, **P < 0.01.

**Table 3:** Effect of age on the association between dietary quality (mean score ± SD) and PAL as well as nutritional knowledge.

	Age groups			*P* value
	Young women (18–25 years)	Women reaching middle age (26–44 years)	Middle-aged women (45–55 years)	
Physical activity level				
Low	15.56 ± 3.39	16.74 ± 3.24	17.30 ± 2.16	0.003**
Moderate	17.58 ± 3.34	17.82 ± 3.36	19.40 ± 2.58	
High	17.98 ± 2.96	17.74 ± 3.03	18.47 ± 2.85	
Nutritional knowledge				
Insufficient nutritional knowledge	10.00 ± 0.00	15.71 ± 2.43	19.00 ± 1.41	0.001**
Quite good nutritional knowledge	16.79 ± 3.20	16.59 ± 3.35	17.76 ± 2.80	
Good nutritional knowledge	17.53 ± 3.41	18.16 ± 3.17	19.19 ± 2.57	

*P < 0.05, **P < 0.01.

**Table 4:** Age group, income level, education level, employment status, marital status and family structure, physical activity level, and nutritional knowledge as determinants of dietary quality.

Variable	Classification	*β*	*P*
Age group	Young	0.172	0.003**
	Reaching middle age		
	Middle aged		

Income level	Low	0.005	0.929
	Moderate		
	High		

Education level	Low	0.129	0.034*
	Moderate		
	High		

Employment status	Student	0.011	0.854
	Self-employed		
	Part time		
	Full time		

Marital status	Single	−0.012	0.853
	Married		
	Divorced/separated		
	Widowed		
	Cohabitation		

Family structure	Nuclear	0.073	0.182
	Extended		

Physical activity level	Low	0.142	0.008**
	Moderate		
	High		

Nutritional knowledge	Insufficient	0.177	0.002**
	Quite good		
	Good		

*P < 0.05, **P < 0.01.

Multiple *R* = 0.317.

Adjusted *R* ^2^ = 0.078.

*F*[8,326] = 4.43, *P* < 0.001.

## References

[1] https://www.cia.gov/library/publications/the-world-factbook/geos/mp.html.

[2] Maurer S (2012). Mauritius: interethnic relations through rice and rum. *Journal of Anthropology*.

[3] Appanah TP, Oogarah-Pratap B, Ruggoo A (2009). Awareness and consumption of iron among Mauritian female factory workers. *Nutrition and Food Science*.

[4] Seechurn D, Neeliah H, Neeliah SA (2009). Functional foods in Mauritius: a consumer survey. *Journal of Development and Agricultural Economics*.

[5] Donner L, Isfeld H, Haworth-Brockman M, Forsey C (2008). A profile of Women's health in manitoba. *Prairie Women’s Health*.

[6] Ransom EI, Elder LK Nutrition of Women and Adolescent Girls: Why It Matters. http://www.prb.org/Articles/2003/NutritionofWomenandAdolescentGirlsWhyItMatters.aspx.

[7] Leong SL, Madden C, Gray A, Horwath C (2012). Self-determined, autonomous regulation of eating behavior is related to lower body mass index in a nationwide survey of middle-aged women. *Journal of the Academy of Nutrition and Dietetics*.

[8] Kimokoti RW, Newby PK, Gona P (2010). Diet quality, physical activity, smoking status, and weight fluctuation are associated with weight change in women and men. *Journal of Nutrition*.

[9] Mishra GD, McNaughton SA, Ball K, Brown WJ, Giles GG, Dobson AJ (2010). Major dietary patterns of young and middle aged women: results from a prospective Australian cohort study. *European Journal of Clinical Nutrition*.

[10] Van Minh H, Ng N, Juvekar S (2008). Self-reported prevalence of chronic diseases and their relation to selected sociodemographic variables: a study in INDEPTH Asian sites, 2005. *Preventing Chronic Disease*.

[11] Kuri-Morales P, Emberson J, Alegre-Díaz J (2009). The prevalence of chronic diseases and major disease risk factors at different ages among 150 000 men and women living in Mexico City: cross-sectional analyses of a prospective study. *BMC Public Health*.

[12] Rizvi SA, Jalil F, Azam SI, Shamsi U, Saleem S (2012). Prevalence of menopause, chronic illnesses and life style of middle aged women in Karachi, Pakistan. *Al Ameen Journal of Medical Sciences*.

[13] McCullough ML, Feskanich D, Stampfer MJ (2002). Diet quality and major chronic disease risk in men and women: moving toward improved dietary guidance. *American Journal of Clinical Nutrition*.

[14] Sharma SV, Gernand AD, Day RS (2008). Nutrition knowledge predicts eating behavior of all food groups except fruits and vegetables among adults in the paso del norte region: qué sabrosa vida. *Journal of Nutrition Education and Behavior*.

[15] Darmon N, Drewnowski A (2008). Does social class predict diet quality?. *American Journal of Clinical Nutrition*.

[16] Irala-Estévez JD, Groth M, Johansson L, Oltersdorf U, Prättälä R, Martínez-González MA (2000). A systematic review of socio-economic differences in food habits in Europe: consumption of fruit and vegetables. *European Journal of Clinical Nutrition*.

[17] Dammann KW, Smith C (2009). Factors affecting low-income Women's food choices and the perceived impact of dietary intake and socioeconomic status on their health and weight. *Journal of Nutrition Education and Behavior*.

[18] Power EM (2005). Determinants of healthy eating among low-income Canadians. *Canadian Journal of Public Health*.

[19] Rberg Kjøllesdal MK, Holmboe-Ottesen G, Wandel M (2010). Associations between food patterns, socioeconomic position and working situation among adult, working women and men in Oslo. *European Journal of Clinical Nutrition*.

[20] Dobson A, Mishra G, Brown W, Reynolds R (1997). Food habits of young and middle-aged women living outside the capital cities of Australia. *Australian and New Zealand Journal of Public Health*.

[21] Fawehinmi TO, Ilomäki J, Voutilainen S, Kauhanen J (2012). Alcohol consumption and dietary patterns: the FinDrink study. *PLoS ONE*.

[22] Prättälä RS, Laaksonen MT, Rahkonen OJ (1998). Smoking and unhealthy food habits: how stable is the association?. *European Journal of Public Health*.

[23] Voorrips LE, Van Staveren WA, Hautvast JGAJ (1991). Are physically active elderly women in a better nutritional condition than their sedentary peers?. *European Journal of Clinical Nutrition*.

[24] Wilcox S, King AC, Castro C, Bortz W (2000). Do changes in physical activity lead to dietary changes in middle and old age?. *American Journal of Preventive Medicine*.

[25] Baghurst KI, Baghurst PA, Record SJ (1994). Demographic and dietary profiles of high and low fat consumers in Australia. *Journal of Epidemiology and Community Health*.

[26] Worsley A (2002). Nutrition knowledge and food consumption: can nutrition knowledge change food behaviour?. *Asia Pacific Journal of Clinical Nutrition*.

[27] Parmenter K, Wardle J (1999). Development of a general nutrition knowledge questionnaire for adults. *European Journal of Clinical Nutrition*.

[28] Wardle J, Parmenter K, Waller J (2000). Nutrition knowledge and food intake. *Appetite*.

[29] Turconi G, Guarcello M, Maccarini L (2008). Eating habits and behaviors, physical activity, nutritional and food safety knowledge and beliefs in an adolescent Italian population. *Journal of the American College of Nutrition*.

[30] Krejcie RV, Morgan DV (1970). Determining sample size for research activities. *Educational and Psychological Measurement*.

[31] Turagabeci AR, Nakamura K, Kizuki M, Takano T (2007). Family structure and health, how companionship acts as a buffer against ill health. *Health and Quality of Life Outcomes*.

[32] Fokeena WB, Jeewon R (2012). Is there an association between socioeconomic status and body mass index among adolescents in mauritius?. *Scientific World Journal*.

[33] Ministry of Health (2000). *Dietary Guidelines for the Prevention of NCDs in Mauritius*.

[34] California Department of Public Health (CDPH) (2012). *MyPlate For Moms: Maternal, Child, and Adolescent Health (MCAH) Nutrition and Physical Activity Initiative*.

[35] Al-Malki JS, Al-Jaser MH, Warsy AS (2003). Overweight and obesity in Saudi females of childbearing age. *International Journal of Obesity*.

[36] Muhihi AJ, Njelekela MA, Mpembeni R, Mwiru RS, Mligiliche N, Mtabaji J (2012). Obesity, overweight, and perceptions about body weight among middle-aged adults in Dar es Salaam, Tanzania. *ISRN Obesity*.

[37] Vahratian A (2009). Prevalence of overweight and obesity among women of childbearing age: results from the 2002 national survey of family growth. *Maternal and Child Health Journal*.

[38] Panagiotakos DB, Chrysohoou C, Siasos G (2011). Sociodemographic and lifestyle statistics of oldest old people (>80 years) living in ikaria island: the ikaria study. *Cardiology Research and Practice*.

[39] Hagströmer M, Oja P, Sjöström M (2006). The International Physical Activity Questionnaire (IPAQ): a study of concurrent and construct validity. *Public Health Nutrition*.

[40] Statistics Mauritius (2013). *Tableau de Bord*.

[41] http://health.gov.mu/English/Documents/ncd-2009.pdf.

[42] Koch V, Pokorn D (1999). Comparison of nutritional habits among various adult age groups in Slovenia. *Nutrition Research*.

[43] Lallukka T, Laaksonen M, Rahkonen O, Roos E, Lahelma E (2007). Multiple socio-economic circumstances and healthy food habits. *European Journal of Clinical Nutrition*.

[44] Yannakoulia M, Panagiotakos D, Pitsavos C, Skoumas Y, Stafanadis C (2008). Eating patterns may mediate the association between marital status, body mass index, and blood cholesterol levels in apparently healthy men and women from the ATTICA study. *Social Science and Medicine*.

[45] Coleman L, Cox L, Roker D (2008). Girls and young women's participation in physical activity: psychological and social influences. *Health Education Research*.

[46] Newton Z, Guo L, Yang H, Malkin M (2012). Physical activity and perception of body image of African American women. *LARNet the Cyber Journal of Applied Leisure and Recreation Research*.

[47] Mama SK, Quill BE, Fernandez-Esquer ME, Reese-Smith JY, Banda JA, Lee RE (2011). Body image and physical activity among Latina and African American women. *Ethnicity & Disease*.

[48] Owens JF, Matthews KA, Wing RR, Kuller LH (1992). Can physical activity mitigate the effects of aging in middle-aged women?. *Circulation*.

[49] Midhet F, Al Mohaimeed AR, Sharaf F (2010). Dietary practices, physical activity and health education in qassim region of Saudi Arabia. *International Journal of Health Sciences (Qassim)*.

[50] Parmenter K, Waller J, Wardle J (2000). Demographic variation in nutrition knowledge in England. *Health Education Research*.

[51] De Vriendt T, Matthys C, Verbeke W, Pynaert I, De Henauw S (2009). Determinants of nutrition knowledge in young and middle-aged Belgian women and the association with their dietary behaviour. *Appetite*.

[52] Herbeth B, Samara A, Stathopoulou M, Siest G, Visvikis-Siest S (2012). Alcohol consumption, beverage preference, and diet in middle-aged men from the STANISLAS study. *Journal of Nutrition and Metabolism*.

[53] Coulston AM, Boushey CJ, Ferruzzi MG (2013). *Nutrition in the Prevention and Treatment of Disease*.

[54] Wrieden W, Peace H, Armstrong J, Barton K (2003). *Report of the Working Group on Monitoring Scottish Dietary Targets: A Short Review of Dietary Assessment Methods Used in National and Scottish Research Studies*.

